# Determination of Rare Earth Elements in Human Sperm and Association with Semen Quality

**DOI:** 10.1007/s00244-015-0143-x

**Published:** 2015-03-12

**Authors:** Urszula Marzec-Wróblewska, Piotr Kamiński, Paweł Łakota, Grzegorz Ludwikowski, Marek Szymański, Karolina Wasilow, Tomasz Stuczyński, Adam Buciński, Leszek Jerzak

**Affiliations:** 1 Department of Biopharmacy, Faculty of Pharmacy, Nicolaus Copernicus University in Toruń, Collegium Medicum in Bydgoszcz, dr. A. Jurasz St. 2, 85-089 Bydgoszcz, Poland; 2 Department of Ecology and Environmental Protection, Faculty of Medicine, Nicolaus Copernicus University in Toruń, Collegium Medicum in Bydgoszcz, M. Skłodowska-Curie St. 9, 85-094 Bydgoszcz, Poland; 3 Department of Biotechnology, Faculty of Biological Sciences, University of Zielona Góra, Prof. Szafran St. 1, 65-516 Zielona Gora, Poland; 4 Department of Animal Biotechnology, Faculty of Animal Biology, University of Technology and Life Sciences, Mazowiecka St. 28, 85-084 Bydgoszcz, Poland; 5 Department of Clinical Andrology, Faculty of Medicine, University Hospital No. 2, Nicolaus Copernicus University in Toruń, Collegium Medicum in Bydgoszcz, Szpitalna St. 19, 85-092 Bydgoszcz, Poland; 6 Department of Obstetrics, Female Pathology and Oncological Gynecology, University Hospital No. 2, Nicolaus Copernicus University in Toruń, Collegium Medicum in Bydgoszcz, Ujejski St. 75, 85-168 Bydgoszcz, Poland; 7 NZOZ Medical Center Co. Prof. dr. hab. med. Wiesław Szymański, Dr. med. Marek Szymański, Waleniowa St. 24, 85-435 Bydgoszcz, Poland; 8 Family Medicine Clinic, University Hospital No. 2, Nicolaus Copernicus University in Toruń, Collegium Medicum in Bydgoszcz, Ujejski St. 75, 85-168 Bydgoszcz, Poland; 9 Department of Soil Structure, Institute of Agriculture and Soil Cultivation, Czartoryskich St. 8, 24-100 Puławy, Poland; 10 Department of Nature Protection, Faculty of Biological Sciences, University of Zielona Góra, Prof. Szafran St. 1, 65-516 Zielona Gora, Poland

## Abstract

The aim of the present study was to measure lanthanum (La), cerium (Ce), europium (Eu), and gadolinium (Gd) concentrations in human semen and correlate the results with sperm quality. The median semen content of La was 19.5 µg kg^−1^ dry weight (dw) (range 2.27–269), of Ce was 41.9 µg kg^−1^ dw (range 4.52 to 167), of Eu was 0.68 µg kg^−1^ dw (range 0.06–1.95), of Gd was 3.19 µg kg^−1^ dw (range 0.38–12.0), and of calcium (Ca) was 4063 mg kg^−1^ dw (range 484–17,191). Concentrations of La, Ce, Eu, Gd, and Ca were significantly lower in nondrinkers’ semen than in semen from drinkers. Significant differences were detected between La, Ce, Eu, Gd, and Ca concentrations in semen from nondrinkers and moderate drinkers. Concentrations of La, Ce, and Gd in semen of short-term smokers were significantly lower than those in extremely long-term smokers. Significant differences were also detected between La concentration in semen from a group of short-term smokers and that of a group of long-term smokers. Positive correlations were found between La, Ce, Eu, Gd, and Ca concentrations in semen. La, Ce, Gd, and Ca concentrations in semen were positively associated with progressive motility and percentage of normal spermatozoa. Positive correlations were found between Ca and sperm concentration. Concentrations of La, Ce, and Gd were negatively associated with sperm concentration, whilst Ca concentration was negatively associated with volume of ejaculate. At the examined level, La, Ce, Eu, and Gd did not affect sperm quality, whereas alcohol consumption and smoking might have increased the level of rare earth elements in semen.

Rare earth elements (REEs) include 17 chemically similar metallic elements: 15 lanthanides plus scandium and yttrium. The elements lanthanum (La), cerium (Ce), europium (Eu), and gadolinium (Gd) belong to this group. During the past few years, the widespread application of rare earth elements has been extensively reported in various fields including agriculture, animal husbandry, electronics, energy, fuel additives, and modern biomedicine. Rare earth ions initially enter organs; after transforming into metastable hydrogen oxide particles, they selectively accumulate in the liver and other reticular structures. This leads to enrichment of REEs in these tissues (Eisele et al. 1980; Nakamura et al. 1997; Chen et al. 2000; Kawagoe et al. 2008; Huang et al. 2011). It is well-known that REEs cause hepatotoxic and neurotoxic effects and lung damage (Basu et al. 1982; Porru et al. 2001; Feng et al. 2006, 2007; Palasz and Czekaj 2000; Kobayashi et al. 2005; Yang et al. 2009). La carbonate has been approved as a medicine for treating renal failure (Yamada et al. 2012), whereas Ce compounds are used for the treatment of severe skin burns and in antioxidant therapy, neuroprotection, radioprotection, and ocular protection (Monafo et al. 1976; Rzigalinski et al. 2006; Korsvik et al. 2007). Several investigators have shown that trivalent lanthanide ions may substitute calcium (Ca) at metal-binding sites in many proteins (Brittain et al. 1976; Martin and Richardson 1979) and membranes (Mikkelson 1976; dos Remedios 1981), whilst Ca is essential for sperm motility (Morton et al. 1974; Tash and Mean 1982; Hong et al. 1984; Fakih et al. 1986; Lindemann et al. 1987; Sørensen et al. 1999; Darszon et al. 2005), metabolic functions (Yanagimachi 1981), and acrosome reaction and fertilization (Yanagimachi and Usui 1974; Yanagimachi 1981; Hong et al. 1984; Sørensen et al. 1999). Only a few examples of REEs affecting semen motility are reported in the literature. Lee et al. (1981) showed that La^3+^ and Eu^3+^ inhibited motility stimulation in the presence of Ca, whereas Saling (1982) showed that sperm immotility in the presence of La^3+^ can be reversed immediately by incubation with Ca^2+^. In addition, Oral et al. (2010) found that both Ce^4+^ and La^3+^ caused a decrease in sperm fertilization rate at a concentration of 10^−5^ M. The REE-related health impacts are still not fully elucidated, and systemic and basic information is still lacking (He and Rambeck 2000; Yu et al. 2007; He et al. 2008, 2010; Yang et al. 2009).

Nearly 40 % of infertility cases are caused by male factor (Vayena et al. 2002); however, most of them are due to the inferior quality or the small quantity of spermatozoa. The rapid development and widespread application of novel REEs technologies in industrialized countries requires additional information on the potential health effects derived from possible exposure to REEs and their compounds. A review of the literature revealed that there is little information regarding the possible impact of REEs on human health, and—to the best of our knowledge—the contents of rare earth elements in human semen have not been determined. Thus, the aim of present study was to first measure La, Ce, Ce, and Ga concentrations in human semen and, second, to correlate the results with sperm quality, total semen concentration of Ca, and lifestyle habits.

## Materials and Methods

### Human Semen

Semen samples were received from male partners of infertile couples (*N* = 168) undergoing routine infertility evaluation at an infertility clinic: “NZOZ Medical Center Co. Prof. dr. hab. med. Wiesław Szymański, Dr. med. Marek Szymański” and “Almed - Genito-Urinary Medicine Clinic - dr G. Ludwikowski.” The inclusion criteria were male partners of infertile couples and attending fertility clinic for fertility evaluation [infertility is “a disease of the reproductive system defined by the failure to achieve a clinical pregnancy after 12 months or more of regular unprotected sexual intercourse” (Zegers-Hochschild et al. 2009)]. All men underwent evaluation, which included medical history, clinical examination, and semen analysis. All participants agreed to participate in the study. The exclusion criteria were occupational exposure to any toxicant, radiation, or heat; or any other reason for their partners’s infertility such as semen anomalies; and the use of any medication with proven influence on male fertility within 3 months before semen analysis. All participants were age between 18 and 50 years, fulfilling inclusion criteria, and no men were excluded.

Each man was interviewed, and a questionnaire was used to receive the following information:

1. *Alcohol use* Information about alcohol use included alcohol consumption status (nondrinkers/drinkers) and amount of alcohol consumed (in grams of alcohol per week. Men answering that they “never’’ used alcohol (0 grams of alcohol/week) were defined as nondrinkers; subjects with a consumption of <150 g alcohol/week were defined as moderate drinkers; and those consuming >150 g alcohol/week were defined as high drinkers.
2. *Tobacco use* Information about tobacco use included tobacco use status (smokers/nonsmokers, short-term/long-term smokers), amount, and duration (in years). Men answering that they “never” used tobacco were defined as nonsmokers. Subjects who had smoked for <1 year were defined as short-term smokers. Those who had smoked from 1 to 5 years were defined as medium-term smokers. Males who had smoked for >5–10 years were defined as long-term smokers. Man who had smoked for >10 years were defined as extremely long-term smokers).

We took account the principles and criteria of the World Health Organization (WHO 2010) procedures for sperm collection, analysis, and definitions in our studies. Thus, after a period of 3–7 days of abstinence, semen samples were collected in sterile containers. After liquefaction, semen analysis was performed according to the WHO guidelines to obtain volume, sperm concentration, motility, and morphology (WHO 2010). Ejaculate volume (ml) was evaluated directly in measuring cylinder (0.1-ml accuracy); sperm concentration (×10^6^ ml^−1^) was determined using a counting chamber; motility (%) was determined at ×400 magnification with phase-contrast optics (200 spermatozoa were counted/semen sample); and percentage of morphologically normal spermatozoa was evaluated using Diff-Quick stain by analyzing 100 spermatozoa/semen sample (sperm morphology was rated according to the WHO guidelines criteria). Normal semen values (according to the WHO criteria) are as follows: volume ≥1.5 ml; sperm concentration ≥15 × 10^6^/ml of semen; motility ≥32 % spermatozoa with progressive motility; and morphologically normal spermatozoa ≥4 % (WHO 2010).

Participants were divided into two groups based on their ejaculate parameters. Group I consisted of healthy male partners of infertile couples with normal ejaculate [normozoospermia; *N* = 83 (unknown fertility)] and also served as the control group. Group II consisted of males identified as having semen anomalies as the only reason for their infertility with abnormal volume of semen, abnormal concentration, morphology, or motility of spermatozoa; males with more than one abnormal semen variables; and males with no spermatozoa in the ejaculate (*N* = 85).

### La, Ce, Eu, Gd, and Ca Analysis in Semen

Seminal La, Ce, Eu, Gd, and Ca were measured in 162 of 168 samples (group I *N* = 80; group II *N* = 82). The method used for determination of La, Ce, Eu, Gd, and Ca concentrations in semen samples is described elsewhere (Marzec-Wróblewska et al. 2011). In brief, 1–1.5 ml of semen sample were evaporated in a mineralizer (Tusnovics firm) first at 105 °C then at 450 °C (14 h). Then 3 ml of 69.0–70.0 % nitric acid (Baker Instra analyzed) was added to each sample. After mixing, samples were located in aluminum mineralizer and heated first at 100 °C (1 h) and then at 150 °C (1 h). The semen samples were poured with 1 ml of H_2_O_2_ (35 %) and heated at 100 °C (1 h). After self-cooling, 6 ml of bidistilled water was added, and a solution was mixed up. La, Ce, Eu, Gd, and Ca concentrations in semen samples were determined using inductively coupled plasma–mass spectrometry (ICP-MS; 7500CE-Agilent ICP-MS; Agilent, Palo Alto, California, USA). Recoveries were assessed trough sample dilution. Five-fold diluted samples showed differences in concentrations compared with the original sample, which was <20 %. The limit of detection for La and Ce was 0.3 µg kg^−1^, for Eu and Gd was 0.3 ng kg^−1^, and for Ca was 0.33 µg kg^−1^. limit of quantification for La and Ce was 0.9 µg kg^−1^, for Eu and Gd was 0.9 ng kg^−1^, and for Ca was 0.33 µg kg^−1^. Precision of the measurements for La, Ce, Eu, Gd, and Ca was 9, 7, 12, 10, and 8 %, respectively. In the determination of Eu, 35 % of samples were lower than the method detection limit (MDL). For Gd, Ce, La, and Ca no results were lower than the MDL.

### Statistical Analysis

The medians were calculated and analyzed using computer program Statistica StatSoft, version 10.0 (StatSoft, College Station, Pennsylvania, USA). The median values of La, Ce, Eu, Gd, and Ca semen concentrations were compared using U-Mann–Whitney and Kruskal–Wallis tests. The relationships between La, Ce, Eu, Gd, and Ca semen concentrations and semen-quality parameters were calculated using Spearman’s rank correlation coefficients, and *p* < 0.05 was regarded as significant. Results lower than the detection limit were excluded from statistical analysis. Data analyses were performed in the three groups of men: a group of all individuals, group I (normozoospermic men), and group II (men with a pathological spermiogram). Ethical approval was obtained for this study from the Local Committee of Bioethics of Nicolaus Copernicus University in Toruń, Collegium Medicum in Bydgoszcz (KB/538/2007).

## Results

### Concentrations of REEs and Ca in Semen

The concentration of La in semen of Polish men was 2.27–269 µg kg^−1^ dry weight (dw) (median 19.5). The semen content of Ce was 41.9 µg kg^−1^ dw (median 4.52–167). Concentration of Ce was the lowest of the measured REEs and was 0.68 µg kg^−1^ dw (median 0.06–1.95). The range of Gd content in semen was from 0.38 to 12.0 µg kg^−1^ dw (median 3.19). In the present study, the range of seminal Ca content was from 484 to 17,191 mg kg^−1^ dw (median 4063). Our study also showed that semen of men from group I had greater La, Ce, and Gd concentrations than semen of men from group II, but the differences were not significant. Median concentration of Eu in semen of group I and II males was similar. The concentration of Ca in semen of group I males was lower than in semen from group II men [the difference was not significant (Table 1)].

**Table 1:** Characteristics of semen in the group of normozoospermic males (group I) and the group of males with abnormal semen values (group II)

Semen characteristics	Group I			Group II			*Z*	*p*
	*N*	Median	Minimum–maximum	*N*	Median	Minimum–maximum		
Ejaculate volume (ml)	83	3.75	1.50–8.00	85	3.50	0.40–10.3	−1.32	0.19
Progressive motility (%)	83	58.8	32.2–84.1	85	12.69	0.00–70.0	**−9.97**	**0.00**
Normal morphology (%)	83	85.7	58.9–99.8	85	63.89	0.00–100	**−5.73**	**0.00**
Sperm concentration (×106/ml)	83	85.5	16.0–260	85	12.80	0.00–343	**−7.60**	**0.00**
La concentration (µg/kg dw)	80	21.5	2.27–269	82	17.5	2.58–79.0	−0.98	0.33
Ce concentration (µg/kg dw)	80	44.0	4.52–140	82	37.9	4.63–167	−1.07	0.28
Eu concentration (µg/kg dw)	52	0.68	0.06–1.54	52	0.68	0.44–1.95	−0.32	0.75
Gd concentration (µg/kg dw)	80	3.38	0.38–12.0	82	2.98	0.44–11.4	−1.12	0.26
Ca concentration (mg/kg dw)	80	3927	484–7303	82	4130	2126–17,191	1.14	0.25

Statistically significant differences are shown in bold type

Comparison of the results of groups of semen samples (*p* < 0.05)

### Alcohol

In the group of all men (group I + II) and group II (males with a pathological spermiogram), concentrations of La, Ce, Eu, Gd, and Ca (Eu and Ca only in group II) was significantly lower in nondrinkers’ semen than in semen from drinkers (Table 2). A significant difference was detected in the group of all men (group I + II) and group II (males with pathological spermiogram) between La, Ce, Gd, and Ca (Gd and Ca also only in group II) concentrations in semen from nondrinkers and that from moderate drinkers (Table 3).

**Table 2:** Concentrations of La, Ce, Eu, Gd, and Ca in semen of drinkers and nondrinkers in the group of normozoospermic males (group I), the group of males with abnormal serum values (group II), and both groups combined

	All individuals			Group I			Group II		
	*N*	Median (minimum–maximum)	*p*	*N*	Median (minimum–maximum)	*p*	*N*	Median (minimum–maximum)	*p*
La concentration (µg/kg dw)									
Nondrinkers	36	16.2^a^ (3.05–5.52)	**0.00**	20	21.1 (4.14–55.2)	0.60	16	8.04^d^ (3.05–21.8)	**0.00**
Drinkers	114	21.8^a^ (2.27–268)		53	24.0 (2.27–269)		61	21.2^d^ (2.58–64.1)	
Ce concentration (µg/kg dw)									
Nondrinkers	36	33.3^b^ (5.37–115)	0.01	20	42.4 (8.97–115)	0.58	16	16.2^e^ (5.37–42.7)	0.00
Drinkers	114	44.3^b^ (4.52–140)		53	49.0 (4.52–140)		61	43.1^e^ (4.63–140)	
Eu concentration (µg/kg dw)									
Nondrinkers	18	0.64 (0.46–1.54)	0.26	13	0.64 (0.52–1.54)	0.85	16	0.54^f^ (0.46–0.71)	0.03
Drinkers	80	0.69 (0.06–1.95)		53	0.68 (0.06–1.34)		61	0.69^f^ (0.44–1.95)	
Gd concentration (µg/kg dw)									
Nondrinkers	36	2.69^c^ (0.44–9.21)	**0.01**	20	3.28 (1.03–9.21)	0.71	16	1.38^g^ (0.44–3.55)	**0.00**
Drinkers	114	3.47^c^ (0.38–12.0)		53	3.79 (0.38–12.0)		61	3.26^g^ (0.50–9.87)	
Ca concentration (mg/kg dw)									
Nondrinkers	36	3842 (2126–7303)	0.12	20	4456 (2590–7303)	0.24	16	2885^h^ (2126–5856)	**0.00**
Drinkers	114	3964 (484–14,617)		53	3734 (484–7070)		61	4318^h^ (2232–14,617)	

Statistically significant differences are shown in bold type

Medians indicated with identical superscript letters are statistically different (*p* < 0.05)

**Table 3:** Concentrations of La, Ce, Eu, Gd and Ca in semen of nondrinkers, moderate drinkers, and high drinkers in the group of normozoospermic males (group I), the group of males with abnormal values of semen (group II), and both groups combined

	All individuals			Group I			Group II		
	*N*	Median (minimum–maximum)	*p*	*N*	Median (minimum–maximum)	*p*	*N*	Median (minimum–maximum)	*p*
La concentration (µg/kg dw)									
Nondrinkers	36	16.2^a^ (3.05–5.52)	**0.01**	20	21.1 (4.14–55.2)	0.84	16	8.04^d^ (3.05–21.8)	**0.00**
Moderate drinkers	102	22.1^a^ (2.27–269)		48	24.7 (2.27–269)		54	21.5^d^ (2.97–64.1)	
High drinkers	12	19.3 (2.58–59.4)		5	22.6 (12.5–41.0)		7	16.7 (2.58–59.4)	
Ce concentration (µg/kg dw)									
Nondrinkers	36	33.3^b^ (5.37–115)	**0.02**	20	42.4 (8.97–115)	0.82	16	16.2^e^ (5.37–42.7)	**0.00**
Moderate drinkers	102	45.0^b^ (4.52–140)		48	50.1 (4.52–140)		54	44.3^e^ (4.63–140)	
High drinkers	12	37.2 (6.03–120)		5	49.0 (23.4–78.7)		7	31.2 (6.03–120)	
Eu concentration (µg/kg dw)									
Nondrinkers	18	0.64 (0.46–1.54)	0.51	13	0.64 (0.52–1.54)	0.92	16	0.54 (0.46–0.71)	0.09
Moderate drinkers	72	0.70 (0.06–1.95)		33	0.68 (0.06–1.34)		39	0.71 (0.04–1.95)	
High drinkers	8	0.65 (0.54–1.41)		5	0.65 (0.01–0.76)		7	0.67 (0.57–0.76)	
Gd concentration (µg/kg dw)									
Nondrinkers	36	2.69^c^ (0.44–9.21)	**0.03**	20	3.28 (1.03–9.21)	0.93	16	1.38^f^ (0.44–3.55)	**0.00**
Moderate drinkers	102	3.61^c^ (0.38–12.0)		48	3.87 (0.38–12.0)		54	3.41^f^ (0.54–9.87)	
High drinkers	12	3.26 (0.50–8.48)		5	3.42 (1.90–5.86)		7	3.25 (0.50–8.48)	
Ca concentration (mg/kg dw)									
Nondrinkers	36	3842 (2126–7303)	0.24	20	4456 (2590–7303)	0.35	16	2885^g^ (2126–5856)	**0.00**
Moderate drinkers	102	3993 (484–14,617)		48	3656 (484–7070)		54	4449^g^ (2455–14,617)	
High drinkers	12	3904 (2232–6853)		5	4209 (2758–6225)		7	3060 (2232–6853)	

Statistically significant differences are shown in bold type

Medians indicated with identical superscript letters are statistically different (*p* < 0.05). Nondrinkers 0 g of ethanol; moderate drinkers ≤150 g of ethanol; and high drinkers >150 g of ethanol

### Tobacco

In the group of all men (group I + II) and group II (males with pathological spermiogram), concentrations of La, Ce, and Gd in semen of short-term smokers were significantly lower than those in semen of extremely long-term smokers (Table 4). A significant difference was also detected in the group of all men (group I + II) between La concentration in semen compared with that from short-term smokers and long-term smokers (Table 4).

**Table 4:** Concentration of La, Ce, Eu, Gd, and Ca in semen of nonsmokers, short-term, medium-term smokers, long-term smokers, and extremely long-term smokers, the group of normozoospermic males (group I), the group of males with abnormal semen values (group II), and both groups combined

	All individuals			Group I			Group II		
	*N*	Median (minimum–maximum)	*p*	*N*	Median (minimum–maximum)	*p*	*N*	Median (minimum–maximum)	*p*
La concentration (µg/kg dw)									
Never smokers	81	18.9 (2.97–75.6)	**0.05** ^**a**^ **0.01** ^**b**^	38	22.5 (4.14–75.6)	0.45	43	16.6 2(.97–53.8)	**0.03**
Short-term smokers	14	9.50^a,b^ (2.27–43.3)		7	9.99 (2.27–43.3)		7	18.2^e^ (7.74–60.2)	
Medium-term smokers	16	16.4 (6.27–269)		7	15.9 (8.66–269)		9	17.0 (6.27–32.7)	
Long-term smokers	24	23.6^a^ (3.51–65.2)		12	19.5 (5.40–55.2)		12	25.0 (3.51–65.2)	
Extremely long-term smokers	21	27.5^b^ (3.02–79.0)		12	24.8 (3.02–46.9)		9	37.1^e^ (7.59–79.0)	
Ce concentration (µg/kg dw)									
Never smokers	81	40.2 (4.63–136)	**0.01**	38	47.5 (8.92–136)	0.47	43	36.2 (4.63–109)	**0.03**
Short-term smokers	14	18.7^c^ (4.52–93.5)		7	22.8 (4.52–93.5)		7	18.2^f^ (7.74–60.2)	
Medium-term smokers	16	33.5 (12.1–140)		7	29.8 (13.0–140)		9	37.1 (12.1–67.5)	
Long-term smokers	24	51.4 (6.03–140)		12	38.9 (9.17–115)		12	55.0 (6.03–140)	
Extremely long-term smokers	21	65.7^c^ (6.04–167)		12	49.4 (6.04–103)		9	82.0^f^ (14.4–167)	
Eu concentration (µg/kg dw)									
Never smokers	53	0.64 (0.15–1.34)	0.31	27	0.65 (0.15–1.34)	0.65	26	0.62 (0.44–1.20)	0.40
Short-term smokers	5	0.78 (0.06–0.90)		3	0.78 (0.06–0.90)		2	0.70 (0.59–0.81)	
Medium-term smokers	9	0.71 (0.54–1.08)		7	0.90 (0.54–1.08)		9	0.69 (0.55–0.75)	
Long-term smokers	16	0.77 (0.49–1.95)		12	0.68 (0.49–1.53)		12	0.77 (0.54–1.95)	
Extremely long-term smokers	17	0.70 (0.53–1.42)		9	0.70 (0.57–1.14)		9	0.76 (0.53–1.42)	
Gd concentration (µg/kg dw)									
Never smokers	81	3.15 (0.44–12.0)	**0.00**	38	3.55 (0.56–12.0)	0.35	43	2.89 (0.44–7.77)	**0.03**
Short-term smokers	14	1.57^d^ (0.38–6.32)		7	1.54 (0.38–6.32)		7	1.60^g^ (0.70–4.85)	
Medium-term smokers	16	2.66 (0.96–7.55)		7	2.56 (1.18–7.55)		9	2.77 (0.96–4.97)	
Long-term smokers	24	3.80 (0.50–9.87)		12	2.84 (1.08–9.21)		12	3.83 (0.50–9.87)	
Extremely long-term smokers	21	4.58^d^ (0.75–11.4)		12	4.15 (0.75–7.10)		9	5.77^g^ (1.05–11.4)	
Ca concentration (mg/kg dw)									
Never smokers	81	3889 (1111–10,305)	0.04	38	3833 (1111–6816)	0.59	43	3889 (2126–10,305)	0.08
Short-term smokers	14	3207 (484–7083)		7	3312 (484–5739)		7	3102 (2232–7083)	
Medium-term smokers	16	3992 (2659–6276)		7	4098 (2659–5517)		9	3886 (2699–6276)	
Long-term smokers	24	4637 (2620–17,191)		12	4042 (2825–7303)		12	5330 (2620–17,191)	
Extremely long-term smokers	21	4809 (1719–14,617)		12	4214 (1719–5863)		9	5138 (3060–14,617)	

Statistically significant differences are shown in bold type

Medians indicated with identical letters are statistically different (*p* < 0.05). Short-term smokers <1 year; medium-term smokers 1–<5 years; long-term smokers 5–<10 years; and extremely long-term smokers ≥10 years

### Correlations

We found a significant positive correlation between La, Ce, Eu, Gd, and Ca concentrations in semen. In the group of all males, concentrations of La, Ce, and Gd in semen were positively associated with progressive motility and percentage of normal spermatozoa. In group I (normozoospermic males), La, Gd and Ca concentrations were also positively associated with progressive motility and percentage of normal spermatozoa, whilst the concentration of Ce was negatively correlated with the percentage of spermatozoa with progressive motility. We also found a significant positive correlation between Ce concentration in semen and percentage of normal spermatozoa and between Ca and sperm concentration. In group II (males with pathological spermiogram), concentrations of La, Ce, and Gd in semen were negatively associated with sperm concentration, whereas Ca concentration was negatively associated with volume of ejaculate (Table 5). Our study show that increased seminal Ca level was associated with increased progressive motility, sperm concentration, and percentage of normal sperm cells.

**Table 5:** Correlations between La, Ce, Eu, Gd, and Ca concentrations in semen and sperm parameters in semen in the group of normozoospermic males (group I), the group of males with abnormal semen values (group II), and both groups combined

Sperm parameters	All individuals					Group I					Group II				
	La	Ce	Eu	Gd	Ca	La	Ce	Eu	Gd	Ca	La	Ce	Eu	Gd	Ca
Ejaculate volume (ml)	−0.00	−0.00	0.04	−0.12	−0.00	0.08	0.07	−0.00	−0.01	0.06	−0.11	−0.09	0.04	−0.22*	−0.09
Progressive motility (%)	0.16*	0.16*	−0.01	0.04	0.16*	0.23*	0.20	−0.28*	0.23*	0.22*	0.05	0.04	−0.01	0.12	0.04
Normal morphology (%)	0.22*	0.22*	0.15	0.11	0.18*	0.28*	0.26*	0.01	0.23*	0.24*	0.12	0.12	0.17	0.13	0.07
Sperm concentration (×10^6^/ml)	−0.01	−0.01	−0.10	−0.04	−0.00	0.14	0.12	−0.18	0.25*	0.13	−0.26*	−0.23*	−0.23	−0.13	−0.23*
La concentration (µg/kg dw)	1.00	0.99*	0.73*	0.71*	0.98*	1.00	0.99*	0.70*	0.70*	0.99*	1.00	0.98*	0.74*	0.74*	0.98*
Ce concentration (µg/kg dw)	0.99*	1.00	0.74*	0.71*	0.99*	0.99*	1.00	0.71*	0.68*	0.99*	0.98*	1.00	0.74*	0.74*	0.98*
Eu concentration (µg/kg dw)	0.73*	0.74*	1.00	0.42*	0.73*	0.70*	0.71*	1.00	0.40*	0.68*	0.74*	0.74*	1.00	0.46*	0.75*
Gd concentration (µg/kg dw)	0.71*	0.71*	0.42*	1.00	0.72*	0.70*	0.68*	0.40*	1.00	0.71*	0.74*	0.74*	0.46*	1.00	0.74*
Ca concentration (mg/kg dw)	0.98*	0.99*	0.73*	0.72*	1.00	0.99*	0.99*	0.68*	0.71*	1.00	0.98*	0.98*	0.75*	0.74*	1.00

Statistical analysis was performed using the Spearman’s rank correlation coefficients (*R*s values are shown in the table)

Statistically significant * *p* < 0.05

## Discussion

To the best of our knowledge, this is the first time that the contents of rare earth elements in human semen has been determined. Concentrations of La, Ce, Eu, and Gd in semen of Polish men were 19.5, 41.9, 0.68, and 3.19 µg kg^−1^ dw, respectively. There are no data concerning this subject in the literature. However, we found some information about concentrations of this element in blood, serum, urine, teeth, and hair. Concentrations of La and Ce in blood plasma of adult males and females living in northern Germany ranged from “not traceable” to 20 ng l^−1^ for La and to 30 ng l^−1^ for Ce (Heitland and Köster 2006). Rodushkin et al. (1999) reported that La and Ce concentrations in serum (3.3 ± 1.7 and 45 ± 24 ng ml^−1^, respectively) were significantly greater than data from a study by Morita et al. (1994), i.e., 0.17 ± 0.2 and 0.40 ± 0.19 ng ml^−1^, respectively. Minoia et al. (1990), who determined concentrations of the elements in urine, blood, and serum (plasma) of the Italian population from 1990, reported that concentrations of La, Ce, and Eu in urine were 0.73 ± 0.55, 3.1 ± 1.95, 0.11 ± 0.08, and <1 µg l^−1^, respectively. The mean values of these elements in blood at the micrograms per liter level were 1.42 ± 0.71 (La), 3.1 ± 2.15 (Ce), 0.21 ± 0.08 (Eu), whereas Gd in urine and La in serum were at concentrations <1 µg l^−1^. Peng et al. (2003) examined the concentration of La in the hair of young children (0–3 years) and their mothers living in a rare earth mining area. The mean hair content of La was greatest (2203 ng g^−1^) in young children living nearest to the REE-mining area. The next greatest (472 ng g^−1^) was in those living nearer to the REEs mining area, and the lowest content (97.4 ng g^−1^) was in those living in the control area. The REE content in the mothers’ hair was 1510 ng g^−1^ in the high-exposure area, 242 ng g^−1^ in the low-exposure area, and 59.2 ng g^−1^ in the control area. Brown et al. (2004) compared La and Ce contents of children’s primary teeth from Uganda and the UK. Teeth from Ugandan children’s contained a significantly greater concentration of La than the teeth in children from the UK (0.001–0.07 and 0.04–0.28 mg kg^−1^ respectively). The range of Ce concentration was greater in teeth in Ugandan children (0.04–0.36 mg kg^−1^) compared with teeth in children from the UK (0.005–0.09 mg kg^−1^). The precise mechanism by which these elements are transferred from the circulatory blood into semen is unclear. In the present study, the median value of seminal Ca content was 4063 mg kg^−1^ dw. Surprisingly, communications in this specific field are also rather limited. Seminal concentration of Ca was reported by Sørensen et al. (1999) as between 391 and 672 mg l^−1^. Valsa et al. (2012) found a Ca concentration in seminal plasma ranging from 8 to 324 mg dl^−1^ and in spermatozoa from 1.58 to 198 mg dl^−1^.

The median values of La, Ce, and Gd concentrations in semen of normospermic men were greater than in semen in men from group II, whereas the concentration of Eu was similar and the concentration of Ca was lower. As previously mentioned, we did not find any information about REE concentrations in semen, but it seems, at the examined level, that La, Ce, Eu, and Gd did not affect sperm quality. However, production, maturation, motility, and fertilizing capacity of the spermatozoa might be affected by abnormal levels of Ca (Hong et al. 1984). Similarly, mean seminal Ca concentration in the fertile group was lower (but not significantly so) than that of the infertile group in research of Wong et al. (2001). This finding also confirms previous reports (Umeyama et al. 1986). Pandy et al. (1983), however, observed a lower concentration of Ca in semen of subfertile males. In addition, Abou-Shakra et al. (1989) determined the lowest Ca content in normozoospermic males of their infertile group. Our results were inconsistent with those reported by Logoglu et al. (1997) and Bassey et al. (2013).

The concentrations of REEs and Ca were lower in nondrinkers’ semen than in semen from drinkers. No data have been reported on REE concentrations in the human body and their relationship with alcohol-consumptions habits, but the results of Xiaofei et al. (2013) indicate that soil containing REEs may be the source of REE pollution in foods, and the human body can continually accumulate REEs through food digestion and absorption. We could not find any information either for and or against our findings on Ca seminal concentration; however the toxic effect of alcohol might manifest, among other things, by a disturbance of chemical element equilibrium in the human body (Ford et al. 1995; Rylander et al. 2001). It is of note that Labib et al. (1989) did not find differences in mean plasma Ca concentration nor any correlation between plasma ethanol and Ca concentration. Similarly, Santori et al. (2008) found no significant difference in total and ionised Ca concentration in serum between chronic male alcoholics and healthy controls. In contrast, Bjørneboe et al. (1986) reported a significantly lower concentration of Ca in serum in alcoholics than in controls. In addition, we found previous reports that ethanol stimulated an increase in intracellular concentration of Ca^2+^ in cells (Davidson et al. 1990; Konda et al. 1991; Kam et al. 2010). Urinary loss of Ca induced by ethanol has also been reported (Lusier et al. 1977). The mechanisms leading to the increase in concentration of REEs and Ca in semen from drinkers are obscure. It must be emphasized that alcohol is not only a chemical substance but also some kind of nutrient and as a nutrient it affects tissue metabolism (Forsander 1998).

Our study shows that concentrations of La, Ce, and Gd in semen are increased with increasing duration of smoking. Data concerning this subject in the literature are scarce. Gerhardsson et al. (1985) and Gerhardsson and Nordberg (1993) found no significant differences in lung tissue La content between smokers, ex-smokers, and nonsmokers, whereas Gómez-Aracena et al. (2006) found a significant relationship between Ce concentration and smoking. It is commonly known that cigarette smoke is a complex mixture of particulate matter and numerous gaseous compounds. However, we could not find good data on La, Ce, and Gd contents in cigarettes. Environmental tobacco smoke (ETS) is one the most relevant and crucial factors for indoor air quality and is classified as being carcinogenic to humans. It contains many trace and toxic elements including heavy metals and rare earth elements. However, for some elements, data are scarce, and little attention has been paid to Ce, La, and Ga contents in ETS. There is no reference to Cd, Ce, La, and Gd in the comprehensive reviews of the International Agency for Research on Cancer (2004) and the United States Department of Health and Human Services (2006). Remarkably, very high concentrations for Ce and La were found in indoor places with moderate or heavy tobacco-smoking activity (Böhlandt et al. 2012). The prevalent source for Ce and La in indoor air appears to be due to tobacco smoke as has already shown by Bolte et al. (2008), Slezakova et al. (2009), and Fromme et al. (2009). As all elements, REEs are present in the atmosphere, water, soil and plants, but, it must be emphasized that REEs are elements with low mobility, and thus they can accumulate in such environments (Cao et al. 2001; Zhang and Shan 2001; Aquino et al. 2009). Thus, long-term intake of small doses of REEs could not be considered as being irrelevant for humans. In addition, human beings are at the top of the food chain; therefore, health damage caused by long-term exposure to REEs from water, food or, e.g., cigarette smoke, should not be neglected.

Our study shows positive correlations between La, Ce, Eu, Gd, and Ca concentrations in semen. Lanthanides act as Ca analogues in biological systems, and trivalent lanthanide ions can replace Ca at metal-binding sites (Wang et al. 1982; Lansman 1990; Nishimura 1993; Reeves and Condrescu 2003; Ardón et al. 2009; Carrillo-López et al. 2010). Seminal La, Ce, and Gd concentrations were positively associated with progressive motility and percentage of normal spermatozoa and negatively with sperm concentration. Ce content was negatively correlated with percentage of spermatozoa with progressive motility. Ca concentration in semen was positively associated with progressive motility, normal morphology, and sperm concentration and negatively associated with volume of ejaculate. Only a handful of reports of the impact of ionized REEs on semen quality were found in the literature. Lee et al. (1981) showed that La^3+^ and Eu^3+^ inhibited motility stimulation in the presence of Ca and that the IC50 values for both ions were approximately 5 × 10^−5^ M. In addition, Saling (1982) reported that sperm immotility in the presence of La^3+^ can be reversed immediately by incubation with Ca^2+^ and, furthermore, that the state of immobilization produced by La^3 +^ did not affect sperm adversely. In Oral et al. (2010), both Ce^4+^ and La^3+^ caused a decrease in sperm fertilization rate at the highest tested concentration (10^−5^ M). Our results do not support this finding; however, it must be emphasized that we measured total concentrations of La, Ce, and Gd in semen and not the effect on spermatozoa of ionized form of this elements. Studies on the relationship between Ca and semen parameters are contradictory. Our study showed that increased seminal Ca levels were associated with increased progressive motility, sperm concentration, and percentage of normal sperm cells. These findings are in accordance with Bassey et al. (2013) who also found significant positive correlation between Ca and percentage motility. However, Arver (1982) showed that a high Ca concentration decreased sperm motility. In contrast, Logoglu et al. (1997) and Wong et al. (2001) found no significant relationships between seminal Ca concentration and sperm density or percentage motility. When our results are evaluated with those of other reports, it may be hypothesized that seminal Ca may be involved in semen quality and that this effect can be stimulatory or inhibitory depending on its concentration. Normal sperm motility, and all other processes leading to fertilization, required an optimal seminal concentration of Ca (Fraser 1987). All of these findings suggest that seminal Ca is an important factor in male fertility.

For the first time, the contents of REEs in human semen were determined and described. Similarly, REE relationships with semen quality, concentration of seminal Ca, and lifestyle habits were reported for the first time. Summarizing our results, although we could not unequivocally prove the influence of La, Ce, Eu, and Gd concentrations on sperm quality as a whole, we found both positive and negative association of La, Ce, Eu and Gd concentrations with semen-quality parameters, i.e., volume of ejaculate, sperm concentration, motility, and morphology. In addition, our findings suggest that alcohol consumption and smoking might increase levels of REEs in semen. However, it is unclear how these elements are transferred into the semen, and a review of the literature reveals that there is little information about the possible impact of REEs on human health. Therefore, further research on La, Ce, Eu, and Gd and the toxicological, human, and public health consequences is urgently required.
